# Significant enhanced expressions of aquaporin-1, -4 and -9 in the brains of various prion diseases

**DOI:** 10.1080/19336896.2019.1660487

**Published:** 2019-09-04

**Authors:** Qi Shi, Yue-Zhang Wu, Xuehua Yang, Kang Xiao, Adalaiti Maimaitiming, Li-Ping Gao, Cao Chen, Chen Gao, Yanjun Guo, Xiao-Ping Dong

**Affiliations:** a State Key Laboratory for Infectious Disease Prevention and Control, Collaborative Innovation Center for Diagnosis and Treatment of Infectious Diseases (Zhejiang University), National Institute for Viral Disease Control and Prevention, Chinese Center for Disease Control and Prevention, Beijing, China; b Department of Neurology, Beijing Friendship Hospital, Capital Medical University, Beijing, China; c Center for Global Public Health, Chinese Center for Disease Control and Prevention, Beijing, China

**Keywords:** Prion disease, aquaporin, astrocyte, scrapie, PrP^Sc^

## Abstract

Aquaporins (AQPs) are widely expressed in various types of tissues, among them AQP1, AQP4 and AQP9 are expressed predominately with relatively special distributing features in various brain regions. The aberrant changes of AQP1 and AQP4 have been observed in the brains of Alzheimer disease (AD). To evaluate the underlying alteration of brain AQPs in prion diseases, scrapie strains of 139A, ME7 and S15 infected mice were tested in this study. Western blots revealed markedly increased levels of AQP1, AQP4 and AQP9 in the brain tissues of all tested scrapie-infected mice collected at terminal stage. Analyses of the AQPs levels in the brain tissues collected at different time-points during incubation period showed time-dependent increased in 139A and ME7-infected mice, especially at the middle-late stage. The AQP1 levels also increased in the cortex regions of some human prion diseases, including the patients with sporadic Creutzfeldt-Jakob disease (CJD), fatal familial insomnia (FFI) and G114V genetic CJD (gCJD). Immunohistochemistry (IHC) assays verified that the AQPs-positive cells were astrocyte-like morphologically; meanwhile, numerous various sizes of AQPs-positive particles and dots were also observable in the brain sections of scrapie-infected mice. Immunofluorescent assays (IFAs) illustrated that the signals of AQPs colocalized with those of the GFAP positive proliferative astrocytes, and more interestingly, appeared to overlap also with the signals of PrP in the brains of scrapie-infected mice. Moreover, IHC assays with a commercial doublestain system revealed that distributing areas of AQPs overlapped not only with that of the activated large astrocytes, but also with that of abundantly deposited PrP^Sc^ in the brain tissues of scrapie murine models. Our data here propose the solid evidences that the expressions of brain AQP1, AQP4 and AQP9 are all aberrantly enhanced in various murine models of scrapie infection. The closely anatomical association between the accumulated AQPs and deposited PrP^Sc^ in the brain tissues indicates that the abnormally increased water channel proteins participate in the pathogenesis of prion diseases.

## Introduction

Prion disease is a group of transmissible neurodegenerative diseases that affect numerous species of mammalian including human being [1,2]. The most representative ones in animals are scrapie in sheep and goat, bovine spongiform encephalopathy (BSE) in cattle and chronic wasting disease (CWD) in elk and deer. Human prion diseases include Creutzfeldt-Jakob disease (CJD), Kuru, Gerstmann–Sträussler–Scheinker syndrome (GSS) and fatal familial insomnia (FFI). The aetiological agent is so-called prion, with the unique characteristics in biology. The propagation of prion is conformational conversion from the cellular prion protein (PrP^C^) to the pathological form (PrP^Sc^) [3]. The neuropathological hallmarks of prion diseases are deposits of PrP^Sc^, spongiform degeneration, neuron loss and reactive astrogliosis in the central nerve system (CNS) [1].

The aquaporins (AQPs) are a family of 13 hydrophobic integral transmembrane water channel proteins, which generally involve in transcellular and transepithelial water movement and fluid transport [4]. The expressions of AQPs are widely observed in various types of tissues and organs. The physiological functions of AQPs cover urine concentration, exocrine gland secretion, hydration of brain, transduction of neuronal signalling and metabolism, etc [5–8]. In the brain tissues, AQP1, AQP4 and AQP9 are expressed predominately with relatively special distributing features in various brain regions [7] and participate in many physiological and pathological processes such as ischaemia, cerebral oedema, epilepsy, K^+^ spatial buffering, development or integrity of blood-brain barrier, synaptic plasticity, spatial memory, and associative fear memory [9–12].

The aberrant changes of AQP1 and AQP4 have been repeatedly observed in the brains of Alzheimer disease (AD) [13–15]. In the postmortem brains of the patients of cerebral amyloid angiopathy (CAA), the level of AQP4 was significantly higher than that of age- and gender-matched controls [16]. Aβ larger number study has verified that perivascular AQP4 localization is significantly associated with AD status independent of age [17]. AQPs are also critical in the clearance of Aβ in the brains of AD. AQP4 is responsible for the clearance of interstitial Aβ from the brain parenchyma in animal experiments [18,19]. AQP1-positive reactive astrocytes modify Aβ deposition in the brain tissues of AD patients [20]. Abnormal increases of AQP1 and AQP4 in the brains of different prion diseases have also described, e.g., the patients of CJD and BSE-infected transgenic mice [21–23].

In this study, the levels of three AQPs isoforms in the brains of the mice infected with scrapie agents 139A and ME7 were evaluated. We noticed remarkable enhanced expressions of AQP1, AQP4 and AQP9 in the brains of scrapie-infected animals at the terminal stage. The increases of brain AQPs in the infected mice occurred much early than the presences of clinical symptoms. Increases of AQP1 were also observed in the postmortem brains of the patients with sporadic CJD (sCJD), FFI and G114V genetic CJD (gCJD). Immunofluorescent assays verified overlapping of AQPs with GFAP-positive cells. Morphological colocalization of AQPs with PrP was also observed.

## Material and methods

### Ethics statement

Usage of the stored brain specimens of human prion diseases and experimental animals in this study was approved by the Ethical Committee of the National Institute for Viral Disease Prevention and Control, China CDC under protocol 2009ZX10004-101. Animal housing and experimental protocols were in accordance with the Chinese Regulations for the Administration of Affairs Concerning Experimental Animals.

### Cell culture

Scrapie agent Chandler-infected cell line SMB-S15 and its normal partner cell line SMB-PS were purchased from Roslin Institute, UK. Cell line SMB-S15-Res was the SMB-S15 cells treated with resveratrol and kept passage for 26 weeks, which was deprived of prion replication *in vitro* and infectivity *in vivo* previously [ref]. Cells were cultured in Dulbecco’s modified Eagle’s medium (DMEM) (Gibco, USA) with 10% foetal bovine serum (FBS) (Ausbian, Australian) at 33°C with 5% CO_2_.

### Experimental scrapie-infected mice

Three different scrapie-infected mouse models were enrolled in this study, including agents 139A-, ME7- and SMB-S15-infected C57BL/6 mice. The clinical and neuropathological characteristics of these models have been described previously [24–26]. The incubation times of 139A-, ME7-, SMB-S15-infected mice were evaluated as 183 ± 23.1, 184.2 ± 11.8, and 172.8 ± 1.8 days, respectively [24]. The SMB-PS-inoculated mice maintained healthy during the observation time (more than 250 days) in the first and second passages. The surgical harvests of the brains of infected animals were performed either under euthanasia with an intraperitoneal injection of chloral hydrate at the moribund time or postmortem after death. Age-matched normal mice were used as the controls. For dynamic study, brain samples of the mice infected with agents 139A and ME7 collected on the 0th, 80^th^, 120^th^, 150^th^ day post-inoculation (dpi) and at terminal stage (representing as 180^th^ dpi) were included.

### Preparation of brain homogenates

Brain tissues were washed in Tris-buffered saline (TBS, 10 mM Tris-HCl, 133 mM NaCl, pH7.4) three times, and then homogenized in lysis buffer (100 mM NaCl, 10 mM EDTA, 0.5% NP-40, 0.5% sodium deoxycholate, 10 mM Tris, pH 7.4) containing protease inhibitor cocktail set III. The homogenates were centrifuged at 2000 × g for 10 min, and the supernatant fractions were aliquoted and stored at −80°C for further experiments.

### Western blots

Aliquots of brain homogenate or cell lysates were separated by 12% SDS-PAGE and electroblotted onto nylon membranes. Membranes were blocked in TBS containing 5% skimmed milk at room temperature (RT) for 2 h and incubated with various primary antibodies at 4°C overnight, including anti-AQP1 antibody (1:500), anti-AQP4 antibody (1:1,000), anti-AQP9 antibody (1:500), anti-β-actin antibody (1:5,000, huaxingbio, HX1827), anti-PrP antibody (1:500, Santa Cruz, sc-58,581). After washing with TBS containing 0.1% Tween 20 (TBST), membranes were incubated with HRP conjugated secondary antibodies (Jackson ImmunoResearch Labs, 115-035-003 and 111-035-003) at RT for 1 h. The blots were developed using an enhanced chemiluminescence system (ECL, PerkinElmer, NEL103E001EA) and visualized on autoradiography films (General Electrics). Images were captured by ChemiDoc ^TM^ XRS + Imager and quantified by Image J software.

To detect the presence of proteinase K-resistant PrP^Sc^, the brain homogenates or cell lysates were digested with a final concentration of 50 μg/ml proteinase K at 37°C for 1 h prior to Western blots.

### Immunohistochemical (IHC) assay

Brain tissue was fixed in 10% buffered formalin solution and paraffin sections (5 μm in thickness) were prepared routinely. Sections were quenched for endogenous peroxidases in 3% H_2_O_2_ in methanol for 10 min before blocked with 5% BSA for 15 min at RT. The sections were incubated with anti-AQP1 (1:50), anti-AQP4 antibody (1:100), anti-AQP9 antibody (1:100), at 4°C overnight. Subsequently, the sections were incubated with HRP-conjugated goat anti-rabbit antibody (Boster, SV0002-12) at 37°C for 1 h and visualized by incubation with 3,3-diaminobenzidine tetrahydrochloride (DAB) (Boster, AR1000). The slices were counterstained with haematoxylin (Boster, AR 0005), dehydrated, and mounted in permount. For detection of PrP^Sc^, the brain slices were exposed to the buffer containing 6M GdnCl at RT for 1 h prior to the routine IHC process.

### IHC with envision G|2 Doublestain system

The presences of AQPs and GAFA or PrP^Sc^ in the brain sections were analysed with commercial EnVision G|2 Doublestain System (Dako) according to the manufacturer’s protocol. The procedure is a sequential double staining where the first antigen is visualized using HRP/DAB+ and the second antigen is visualized using AP/Permanent Red. All specified reagents, except the primary antibodies are provided with the kit. Briefly, after blocking of endogenous enzymes, the brain sections were incubated with anti-GFAP (1:100) at 4°C overnight, followed by incubation with the Polymer/HRP reagent which contained an HRP-conjugated dextran polymer carrying antibodies to mouse and rabbit immunoglobulins. The reactions were visualized by DAB + Chromogen. Following a blocking step using the Doublestain Block reagent, the sections were separately incubated with anti-AQP1 (1:50), anti-AQP4 antibody (1:100) or anti-AQP9 antibody (1:100) at 4°C overnight. Then, Rabbit/Mouse (Link) was added which was a dextran polymer carrying antibodies to mouse and rabbit immunoglobulins, followed by incubation with the Polymer/AP reagent. Permanent Red Chormogen visualized the second reaction. For evaluating the presences of PrP^Sc^ and AQPs, the brain sections were firstly exposed to the buffer containing 6M GdnCl at RT for 1 h, and then, subjected into the process of the double-staining system by incubation with anti-PrP mAb 6D11 (1:100) at 4°C overnight.

### Immunofluorescence (IF) assay

Brain sections treated with 0.3% Triton-X100 for 30 min and blocked with 5% BSA for 1 h at RT. The sections were incubated with primary antibodies, including anti-AQP1 (1:50), anti-AQP4 antibody (1:100), anti-AQP9 antibody (1:100), anti-PrP antibody (6D11, 1:300, Santa Cruz, Sc-58,581), anti-GFAP antibody (1:300, CST, #3670), at 4°C overnight. Subsequently, brain sections were incubated with Alexa Fluor® 488-conjugated goat anti-rabbit antibody (1:200, Thermo, A-11,008) or/and Alexa Fluor® 568-conjugated goat anti-mouse antibody (1:200, Thermo, A-11,004) at RT (brain sections at 37°C) for 1 h. After stained with 1 μg/mL 4ʹ6-diamidino-2-phenylindole (DAPI, Beyotime, China) for 30 min, the slices were mounted with Permount and viewed using Operetta (Perkinelmer) or Olympus FV1000 confocal microscopy.

### Statistical analysis

Quantitative analysis of immunoblot images was carried out using software Image J. The grey values of each target blot were evaluated. Quantitative evaluations of fluorescent intensity in IFA assays were automatically performed with the software Columbus in Operetta. Statistical analyses were performed using Student’s t-test.

## Results

### Remarkable increases of AQP proteins in the brain tissues of scrapie-infected mice and human prion diseases at the terminal stage

**Figure 1.**
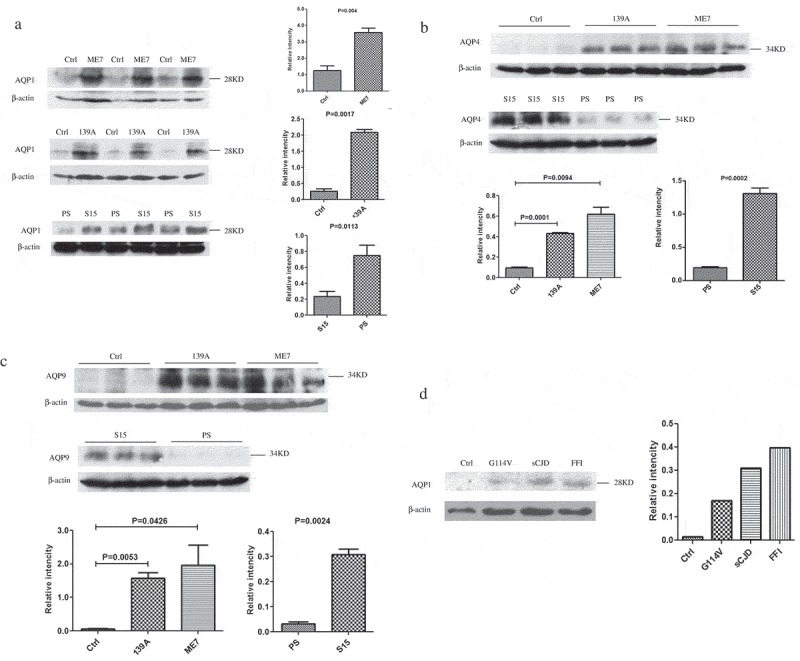
Increased levels of AQP1, AQP4 and AQP9 in the brain tissues of various prion diseases. The levels of three AQP isoforms in the brain homogenates were evaluated by Western blots with the individual antibodies. (a). AQP1 in the brains of scrapie-infected mice. (b). AQP4 in the brains of scrapie-infected mice. (c). AQP9 in the brains of scrapie-infected mice. Three mice infected with scrapie agent 139A, ME7 or S15 were employed, while three age matched mice and three mice inoculated with the lysate of SMB-PS cells were used as control. (d). AQP1 in the homogenates from frontal lobes of various human prion diseases, including sCJD, FFI and G114V-gCJD. β-actin was used as an internal control. The densities of signals are determined by densitometry and showed as AQP/β-actin after normalized with the individual values of β-actin. Graphical data denote mean+SD. Statistical differences compared with controls are illustrated on the top.

To assess the potential changes of AQP proteins in the brains of prion diseases, the brain homogenates of various scrapie-infected mouse models at the terminal stage were prepared and subjected into AQP1, AQP4 and AQP9-specific Western blots, respectively. Compared with that of age-matched normal mice, much stronger AQP1 signals, migrating around 28 to 35 KD in SDS-PAGE, were detected in the preparations of 139A and ME7-infected mice, showing the significant difference after quantitative assays of the grey values with the individual grey of β-actin (Figure 1(a)). Similarly, the brain AQP1 levels in the mice infected with the lysates of SMB-S15 cells were markedly higher than that of the mice inoculated with the lysates of the normal cell line SMB-PS, with a statistical difference (Figure 1(a)). Likewise, AQP4 (Figure 1(b)) and AQP9 (Figure 1(c)) specific Western blots revealed that the levels of those two proteins in the brain tissues of 139A and ME7-infected mice were significantly increased. Futhermore, the levels of AQP1 in the brains of human prion diseases were evaluated. Compared with the data of the sample of normal control prepared from the brain donated by a person died of car accident, AQP1 levels in the brains of a G114V-gCJD, a sCJD and a D178N-FFI pateint were increased (Figure 1(d)). It illustrates an obvious increase of brain AQP proteins in various types of prion diseases at the terminal stage.

### Continual increases of AQP proteins in the mice brain tissues during scrapie infections

**Figure 2.**
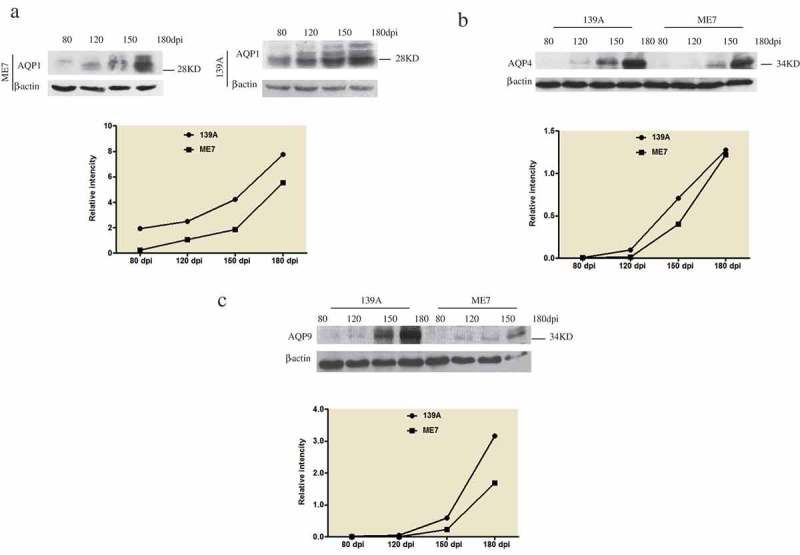
Dynamic alterations of AQPs in the brains of the mice infected with scrapie agents 139A and ME7. A. AQP1. B. AQP4 C. AQP9. The tested samples of 80, 120, 150 and 180 dpi were pooled from the brains of three individual infected mice collected at different time points. The levels of AQPs were tested by the individual specific Western blots. The densities of signals are determined by densitometry and showed as AQP/β-actin after normalized with the individual values of β-actin.

To test the dynamic changes of the brain AQP proteins during prion infection, the brain samples of 139A and ME7-infected mice collected at the different time-points during incubation times (80, 120 and 150 dpi) and at terminal stage (180 dpi) were subjected into Western blots. The sample representing each time-point was prepared by a mixture of the brain homogenates of three individual mice. As shown in Figure 2, all three tested AQP proteins, AQP1 (2A), AQP4 (2B) and AQP9 (2C), showed the similar pattern of time-dependent increase in the brains of both 139A- and ME7-infected mice. The increases of the levels of brain AQP proteins, especially AQP4 and AQP9, became much more remarkable in the samples of 150 dpi and reached to the top in those of 180 dpi.

### More deposits of AQP proteins in the brain slices of scrapie-infected mice at the terminal stage

**Figure 3.**
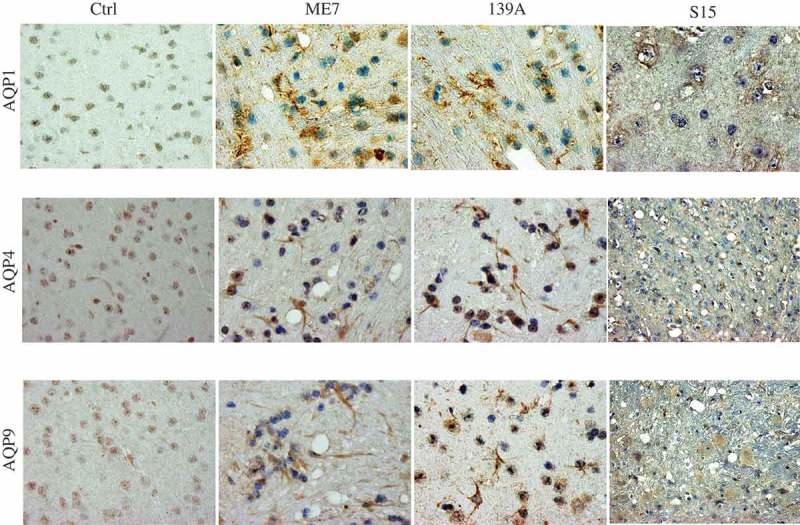
Accumulations of AQPs-specific signals in the brain slices of 139A- and ME7-infected mice. Representative images of AQP1, AQP4 and AQP9 in the cortical regions of the infected mice at terminal stage and age-matched normal ones in IHC assays. (×100).

To get more evidences of up-regulations of brain AQP proteins in prion infection, the brain slices of 139A- and ME7-infected mice at the terminal stage were immunohistochemically stained with the antibodies for various AQP proteins. Compared with the graphs of normal mice, the specific brown signals of AQP1, AQP4 and AQP9 in the cortex regions of both 139A- and ME7-infected mice were much obvious, showing more deeply staining and more widely distribution (Figure 3). Carefully analysis of the stained brain slices illustrated that the AQP4 and AQP9 positive signals positive signals in the preparations of scrapie-infected mice displayed astrocyte-like and fibrous morphologically with relatively event colour, whereas the AQP1 signals appeared various sized granular deposits inside or outside of cells (Figure 3). Particularly, there were numerous deposits of AQPs around the vacuoles in the brains of the infected mice.

### Colocalizations of AQP proteins with GFAP-positive cells in the mice brain tissues

**Figure 4.**
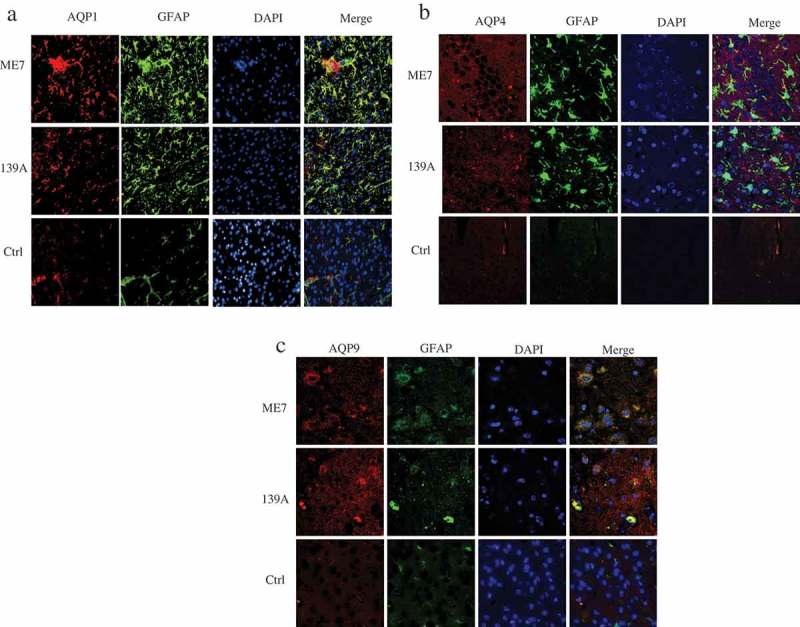
Morphological overlapping of AQPs-specific signals with GFAP-positive cells in the brain slices of 139A- and ME7-infected mice. (a). Representative images of AQP1 (red) with GFAP (green). (b). Representative images of AQP4 (red) with GFAP (green). (c). Representative images of AQP9 (red) with GFAP (green). The brain slices of age-matched normal mice were used as the control. The images were monitored and captured by high content screening system (Operetta Enspire). (×40).

AQP proteins are considered to be predominantly expressed in astrocytes in brain tissues. To look into the location of the increased AQP proteins in the brains of scrapie-infected animals, the brain sections of 139A- and ME7-infected mice were employed into double-stained IFA tests, together with that of normal control. The assays of the double-stained IFA with AQP1 antibody together with GFAP antibody showed a large amount of colocalization of AQP1 singals (red) with GFAP-positive cells (green), particularly in the brain slices of scrapie-infected mice (Figure 4(a)). Markedly more amounts of AQP4 and AQP9 signals were also detected in the brain slices of scrapie-infected animals, showing overlapping with the increased GFAP signals (Figure 4(b, c)). It implies the increased brain AQPs in the scrapie-infected mice locate mainly in the proliferating astrocytes.

**Figure 5.**
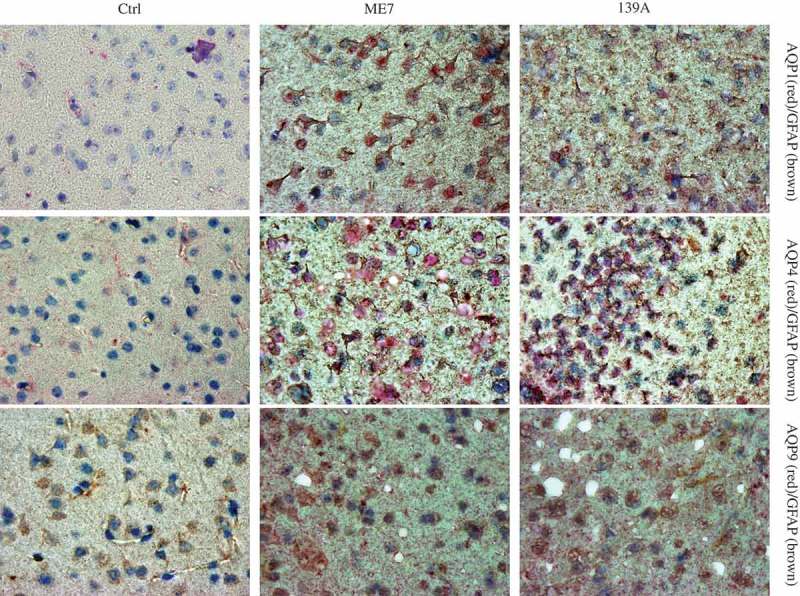
Co-distribution of AQPs signals with GFAP signals in the brain slices of 139A- and ME7-infected mice. Representative images of GFAP signals visualized by incubation with DAB (brown) and AQPs signals developed by incubation of permanent red (red). The brain slices of age-matched normal mice were used as the control. The preparations reacted with the antibodies of AQP1, AQP4 and AQP9 are showed in upper, middle and low panels, respectively. (×100).

To see the distributions of three AQPs and GFAP signals in the brains of scrapie-infected mice, the brain slices of 139A- and ME7-infected mice and age-matched normal mice were subjected into IHC assay with a commercial Doublestain System. All slices were stained with GFAP specific antibody and developed by DAB firstly, and then, reacted with individual AQP specific antibodies and developed by permanent red. As shown in Figure 5, only remarkably less and weak GFAP- (brown) and AQPs-specific signals distributed in the brain slices of normal mice. Contrarily, numerous strongly stained red (AQPs) and brown (GFAP) signals spread all over the brain tissues of 139A- and ME7-infected mice, accompanied with amounts of vacuoles. Astrocyte-like positively stained signals, which were red and/or brown coloured, were observed in the preparations of AQP1 (upper panel), AQP4 (middle penal) and AQP9 (low panel).

### Deposits of AQP-positive signals with PrP signals in the mice brain tissues

**Figure 6.**
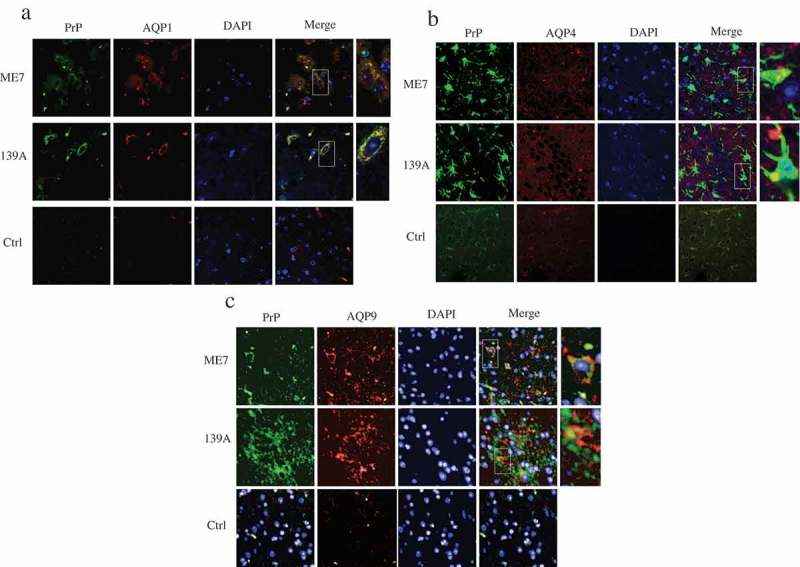
Morphological overlapping of AQPs-specific signals with PrP positive signals in the brain slices of 139A- and ME7-infected mice. (a). Representative images of AQP1 (red) with PrP (green). (b). Representative images of AQP4 (red) with PrP (green). (c). Representative images of AQP9 (red) with PrP (green). The brain slices of age-matched normal mice were used as the control. The images were monitored and captured by confocal microscopy. (×100).

To test the association between the increased AQPs and deposits of PrP in the brains of scrapie experimental rodents morphologically, the brain slices of 139A- and ME7-infected mice were subjected into AQP/PrP double-stained IFA. Confocal microscopy revealed notably stronger signals of both AQP1 (red) and PrP (green) in the preparations of scrapie experimental mice than that of normal control (Figure 6(a)). After merging the images, large amounts of yellow signals were observed inside of the cells. Double-stained IFA with AQP4 (red) and PrP (green) showed yellow overlapped particles insides of the AQP4 positive astrocytes in the brain slices of 139A- and ME7-infected mice (Figure 6(b)). Similarly, overlapped AQP9/PrP signals were also detectable in the brain sections of scrapie-infected mice (Figure 6(c)). It indicates the increased AQPs and PrP in the rodent scrapie brains closely associated each other morphologically.

**Figure 7.**
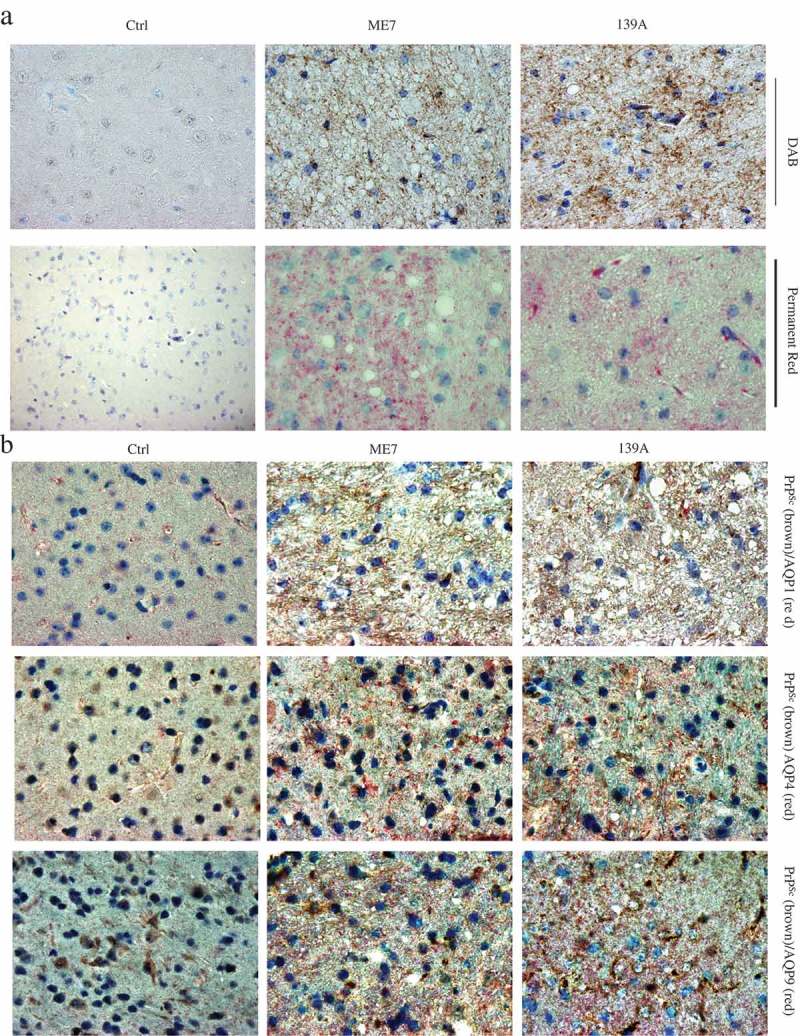
Co-distribution of AQPs signals with PrP^Sc^ signals in the brain slices of 139A- and ME7-infected mice. (a). Representative images of PrP^Sc^ deposits visualized by incubation with DAB (brown) or permanent red (red). The brain slices of age-matched normal mice were used as the control. (b). Representative images of PrP^Sc^ signals visualized by incubation with DAB (brown) and AQPs signals developed by incubation of permanent red (red). (×100). The brain slices of age-matched normal mice were used as the control. The preparations reacted with the antibodies of AQP1, AQP4 and AQP9 are showed in upper, middle and low panels, respectively. (×100).

Further, the morphological association between AQPs and PrP were evaluated by IHC assays using Doublestain System. Prior to double staining, the deposits of PrP^Sc^ in the brains of scrapie-infected animals were evaluated by mAb 6D11-specific IHC tests visualized either by DAB or by permanent red, after removal of normal PrP^C^ by exposure to 6M GdnCl. As shown in Figure 7(a), lager amounts of fine brown (developed by DAB, upper panel) or red (developed by permanent red, low panel) particles distributed in the brain slices of 139A- and ME7-infected mice. The morphological patterns of PrP^Sc^ signals developed by DAB and permanent red were similar. No PrP^Sc^-specific signal was observable in the brain slices of normal mice.

To conduct the double staining with Doublestain System, the brain slices were firstly treated with 6M GdnCl and incubated with PrP-specific mAb 6D11, and subsequently reacted with the antibodies against different AQPs, respectively. Massive brown (PrP^Sc^) and red (AQPs) particles with different sizes were observed in the brain tissues of 139A- and ME7-infected mice, whereas only small amount of red (AQPs) signals were detectable in the slices of normal mice (Figure 7(b)). The distributing area of PrP^Sc^ appeared to overlap with that of various AQPs in the brain tissues with various sized vacuoles.

## Discussion

As water channel proteins, APQs distribute overall in various tissues and organs [27]. In CNS tissues, AQP1, AQP4 and AQP9 are predominant, functioning various physiopathological activates [28,29]. Our previous studies of global protein analysis with proteomics have proposed increased expressing profiles of AQPs in the brain tissues of different human prion diseases and murine models of scrapie infection [30–32]. In this study using different methodologies, we have demonstrated the overexpressions of AQP1, AQP4 and AQP9 in the brains of several models of scrapie-infected mice during incubation times and at the terminal stage. The study here, coincidental with other previous studies [21–23], illustrates aberrant expressions and accumulations of the family of water channel proteins in CNS in prion diseases.

The incubation periods of three scrapie agents on the experimental mice are similar, displaying apparent clinical manifestations, e.g., trembling, sluggishness, ataxia and weight loss, approximately 180 days after intracerebral inoculation. Marked overexpressions of three AQP proteins in brain tissues of scrapie-infected mice occur earlier than the onset clinically. Rapid increases of all three AQPs accumulations in the brain tissues seem to occur at the middle-late stage of the disease, in which the typical neuropathological signs of prion disease are also rapid presented, such as PrP^Sc^ deposit, spongiform degeneration, gliosis and neuron loss in the tested murine models [25,26]. Significant increases of brain AQP1 and AQP4 are also observed in BSE-infected bovine-PrP transgenic mice at the final stage of disease, but not in the early and middle stage [23]. Actually, numerous abnormally changed proteins, regardless of up- or down-regulated, show the similar time-dependent alternative trends, which become more significant at the middle-late stage of prion infection [33–38], reflecting a gradual but persistent progression of neuropathology in prion disease.

Our IHC and IFA data herein illustrates that all tested three AQPs colocalize well with GFAP positive cells in brain tissues. Morphologically, the cells positively stained by three different AQP antibodies display astrocyte-like structures. Meanwhile, remarkably less overlapping signals are seen between AQPs and neurons in the brain tissues (data not shown). In the CNS tissues, AQP1 is reported to distribute mainly in brain choroid plexus and colocalize with Na^+^-, K^+^-ATPase [39]. AQP4 locates at the perimicrovessel astrocyte foot processes, glialimitans and ependymal [40,41]. AQP9 is expressed in astrocytes, cerebellar neurons, and pial vessel endothelium [42]. Although those three AQPs are encoded by the individual-specific genes, which locate at different chromosomes, i.e., *AQP1* in chromosome 7, *AQP4* in chromosome 18 and *AQP9* in chromosome 15, they show similar molecular weights and structures composed of a bundle of six transmembrane α-helices [29]. More importantly, numerous evidences have verified that astrocyte is the main resource for expressing APQ proteins in CNS system [27,29]. Our data here are coincidental with many other previous reports and indicate again that those increased brain AQPs of scrapie-infected mice are expressed by the activated astrocytes. However, the mechanism of astrocytes proliferation and increase of brain AQPs remains unclear. Meanwhile, spongiform change is likely the result of abnormal membrane homoeostasis and increased water content within swollen cell processes. Therefore, the exploration of mechanisms regulating water homoeostasis seems to be a promising step towards the understanding of the neuropathology of prion disease.

Our IHC and IFA assays here have proposed the morphological colocalizations of three AQPs with PrP, even with PrP^Sc^ in the brains of scrapie-infected mice. Moreover, besides of the astrocyte-like strained signals of AQPs, massive AQPs-positive small particles and dots are also observed. Those small AQP-associated particles locate close to the PrP^Sc^ signals anatomically. AQP1 and AQP4 are known to be abnormally expressed in AD brains. Hoshi and the colleagues have described that AQP1 and AQP4 positive cells locate close to Aβ42- or Aβ40-positive senile plaques in the brains of AD patients and speculated that AQP1-positive reactive astrocytes may modify Aβ deposition in the AD brain, while the Aβ deposition process might alter the astrocytic expression of AQP4 [20]. Unlike the observations in AD brains, typical amyloid plaques in brains are observable in small portion of human prion diseases, such as variant CJD and GSS [43]. In the scrapie mice models used in this study, the pathological PrP^Sc^ are extensively dispersive deposits in brain tissues. It is interesting to know whether AQPs-positive astrocytes also locate around or close to the PrP amyloid plaques.

Although the aberrant expressions of brain AQPs let people speculate that they may account for an imbalance in water and ion homoeostasis in prion diseases, the exact molecular mechanism is still scanty. One of the histological hallmarks in the brains of prion disease is spongiform change [43]. The three AQPs-positive astrocytes or signals in this study are frequently observed to locate close to, and even surround different-size vacuoles. Whether and how the increased AQPs contribute to the formation of spongiform degeneration deserves further explored.

Activation status for both astrocytes and microglia along with secretion of cytokines and chemokines should be considered for neuroinflammation [44]. During this process, increases of AQPs are observed not only in the acute phase after brain injury along with oedema, but also in chronic neurodegeneration diseases including prion diseases. AQPs, especially AQP4, are implicated in proinflammatory features of astrocytes, which is an aggravating factor in the AD pathology [45]. AQP1 is believed to involve in cleaning out Aβ aggregation [20]. Attenuating astrocyte activation has been shown to accelerate plaque pathogenesis in mouse AD model [46]. Deletion of AQP4 reduces Aβ-induced activation of cultured astrocytes, which is associated with a reduction in the uptake of Aβ [47]. Knockout of *AQP4* (AQP4^−/−^) in Aβ precursor protein/presenilin 1 (APP/PS1) transgenic mice not only reduces neuroinflammation, but also aggravates brain Aβ accumulation, subsequently exacerbating synaptic protein loss, astrocyte atrophy and cognitive dysfunction [18]. Up-regulation of AQP is also observed in the brains of the patients with Parkinson disease (PD). Immunohistochemical assay has proposed that there is a significant negative correlation between the levels of AQP4 and α-synuclein in layers V-VI, and between those of AQP1 and α-synuclein in layers II-III of PD brains [48]. However, another study has showed that AQP4 KO mice exhibit increased NF-κB activity and enhanced gliosis (astrocytosis and microgliosis) in chronic MPTP (1-methyl-4-phenyl-1,2,3,6-tetrahydropyridine)/probenecid PD models, companying with the increase in the production of IL-1β and TNF-α in the midbrain [49]. Whether the increase of neuroinflammation via AQP4 knockout in the PD mice is model special remains unknown. Nevertheless, the marked enhanced various AQP proteins in brain tissues of AD, PD and prion diseases strongly reflect an active neuroinflammation in those neurodegeneration disorders, which probably aims to uptake or eliminate the accumulations of the pathological proteins.
